# 517 Continuous Post-Pyloric Tube Feeds During Prone Operations Do Not Significantly Increase Rates of Pneumonia

**DOI:** 10.1093/jbcr/irae036.152

**Published:** 2024-04-17

**Authors:** Kristiana Sather, Alexandra M Lacey

**Affiliations:** Regions Hospital, Minneapolis, MN; Regions Burn Unit, Saint Paul, MN; Regions Hospital, Minneapolis, MN; Regions Burn Unit, Saint Paul, MN

## Abstract

**Introduction:**

It is well established that nutrition is critical for burn patients due to their catabolic, hypermetabolic, and inflammatory states, and enteral nutrition is the preferred route. For patients with significant burns and those who are unable to meet caloric demands, tube feeds are the preferred method of nutrition. It is important to minimize interruptions in nutrition and in collaboration with the anesthesia colleagues at our institution patients continue to receive continuous post-pyloric tube feeds intraoperatively regardless of positioning. We hypothesized that the rates of post-operative pneumonia would not increase despite receiving post-pyloric tube feeds while in prone positioning.

**Methods:**

This retrospective cohort study includes all pediatric and adult burn patients admitted to our institution between January 2022 through July 2023. The primary outcome was the rate of post-operative pneumonia in patients receiving continuous tube feeds during prone positioning in the operating room compared to the rates of pneumonia in all admitted burn patients. Pneumonia is defined as pneumonia discussed at our Morbidity and Mortality conference and in the continuous tube feeds during prone positioning group, this was further defined as being documented within 48 hours post-operatively. The secondary outcome is the rate of post-operative pneumonia following all prone operative cases in which patients received continuous post-pyloric tube feeds. A p value < 0.05 is considered statistically significant.

**Results:**

Of 855 burn admissions during this time frame, 46 patients received continuous post-pyloric tube feeds while in prone positioning during at least one trip to the operating room. Of these 46 patients, 4.3% developed post-operative pneumonia compared to a 2.2% rate of pneumonia in all admitted burn patients (p = 0.178). Considering some patients required multiple operations, there were a total of 94 operative cases involving patients that received continuous post-pyloric tube feeds while prone and the rate of post-operative pneumonia when these cases were analyzed as individual events was 2.1%.

**Conclusions:**

Although continuous post-pyloric tube feeds during prone cases in the operating room was associated with an increase in pneumonia compared to all burn patients, this was not statistically significant. Furthermore, when the rate of post-operative pneumonia was stratified by total number of prone cases rather than by number of patients, the rate of pneumonia decreased to the same rate of pneumonia in the general burn population.

**Applicability of Research to Practice:**

The purpose of this project is to promote uninterrupted nutrition in burn patients and encourage other institutions to consider continuing post-pyloric tube feeds during prone operating room positioning.

